# Development and Validation of Tetranucleotide Repeat Microsatellite Markers at the Whole-Genome Level in the Yangtze Finless Porpoise

**DOI:** 10.3390/ani15172603

**Published:** 2025-09-04

**Authors:** Mengting Tang, Denghua Yin, Jianglong Que, Danqing Lin, Congping Ying, Jie Liu, Fangning Liu, Pan Wang, Wenwen Li, Jinxiang Yu, Kai Liu

**Affiliations:** 1 National Demonstration Center for Experimental Fisheries Science Education, Shanghai Ocean University, Shanghai 201306, China; m230150512@st.shou.edu.cn (M.T.); m240100178@st.shou.edu.cn (W.L.); 2 Key Laboratory of Freshwater Fisheries and Germplasm Resources Utilization, Ministry of Agriculture and Rural Affairs, Freshwater Fisheries Research Center, Chinese Academy of Fishery Sciences, Wuxi 214081, China; yindenghua@ffrc.cn (D.Y.); lindq@ffrc.cn (D.L.); yingcongping@ffrc.cn (C.Y.); maydayjiel@163.com (J.L.); wangpan@ffrc.cn (P.W.); 3 Aquatic Conservation and Rescue Center of Jiangxi Province, Nanchang 330096, China; que_jianglong@sina.com (J.Q.); 15390888556@163.com (F.L.)

**Keywords:** Yangtze finless porpoise, microsatellite, tetranucleotide, genome, genetic diversity assessment

## Abstract

The Yangtze finless porpoise (*Neophocaena asiaeorientalis asiaeorientalis*, YFP) is the only freshwater cetacean species currently found in China’s Yangtze River. To accurately evaluate its genetic diversity and provide reliable molecular markers for population genetic studies, this study developed a highly efficient and reproducible method for identifying polymorphic microsatellite loci using whole-genome sequencing data. Using this method, we identified and validated a set of highly polymorphic microsatellite markers, which were then used to analyze the genetic diversity of the YFP populations in Poyang Lake to evaluate their effectiveness. Our results demonstrated that the screening pipeline successfully identified 220 tetranucleotide repeat microsatellite loci. Based on the principle of uniform chromosomal distribution, 190 loci were randomly selected for experimental validation, of which 19 exhibited stable amplification, high polymorphism, and a low genotyping error rate. Genetic diversity analysis based on these markers revealed significant genetic variation among YFP populations in Poyang Lake, confirming the effectiveness of the developed markers. The polymorphic microsatellite molecular marker system developed in this study demonstrates high reliability and applicability for assessing YFP genetic diversity. This system provides a critical technical foundation for future research in conservation genetics, genetic resource preservation, and the development of genetic management strategies for the species.

## Simple Summary

The Yangtze finless porpoise (*Neophocaena asiaeorientalis asiaeorientalis*) serves as a key flagship species for evaluating the effectiveness of fishing bans and assessing the ecological health of the Yangtze River ecosystem, China. To provide stable and reliable molecular markers for population genetic studies, this study utilized high-quality chromosome-level genomic data of the Yangtze finless porpoise along with resequencing data from 12 individuals to identify highly variable microsatellite loci. By integrating PCR amplification with capillary electrophoresis technology, this study successfully validated a total of 19 tetranucleotide repeat microsatellite loci that exhibit stable amplification, high polymorphism, and low genotyping error rates. Using these molecular markers, a genetic diversity analysis was performed on the Poyang Lake population to assess their functional advantages and applicability. The microsatellite markers developed in this study serve as a critical tool for future accurately identifying genetic relationships among Yangtze finless porpoises and reliably predicting population dynamics.

## 1. Introduction

The Yangtze finless porpoise (*Neophocaena asiaeorientalis asiaeorientaliss*, YFP) belongs to the order Cetacea, suborder Odontoceti, family Phocoenidae, and genus *Neophocaena* [1]. As an endemic small odontocete species in China, it is primarily distributed in the middle and lower reaches of the Yangtze River, as well as in Dongting Lake and Poyang Lake [2,3]. Since the late 20th century, rapid socioeconomic development within the Yangtze River Basin has substantially increased human activities, leading to progressive degradation of aquatic ecosystems and a significant decline in aquatic biodiversity [4]. In 2018, whole-genome resequencing confirmed that the YFP is an independent species [5]. In 2013, the species was classified as “Critically Endangered” (CR) by the International Union for the Conservation of Nature’s Species Survival Commission (IUCN/SSC). On 5 February 2021, the revised “List of National Key Protected Wild Animals” upgraded the conservation status of the YFP to that of a national first-class protected species [6]. According to a scientific survey in 2022, the estimated population size was approximately 1249 individuals, representing a 23.4% increase compared to 2017. Although this marks a notable recovery from previous population declines, the total number remains critically low, and the species’ endangered status persists. Therefore, urgent conservation efforts are still required [7]. To accurately assess the current status of YFP genetic resources, it is crucial to investigate its population genetic characteristics from a molecular perspective. Studies in population genetics enable a precise evaluation of genetic diversity of the YFP, elucidate the population genetic structure, and quantify genetic differentiation across geographically distinct populations. A comprehensive understanding of these key genetic parameters will serve as a scientific foundation for formulating evidence-based conservation and management strategies based on genetic data.

Microsatellite DNA, also known as simple sequence repeats (SSRs), consists of tandemly repeated DNA sequences composed of 1 to 6 nucleotides. Owing to its characteristics of high polymorphism, co-dominant inheritance, and reliable repeatability in detection, microsatellite DNA has become one of the most widely utilized molecular markers in the field of population genetics [8]. In the conservation of endangered species, microsatellite markers are extensively applied to assess genetic diversity, determine kinship relationships, analyze population genetic structure, and investigate evolutionary history. These applications provide a key molecular basis for the formulation of scientifically grounded conservation strategies [9,10]. However, research on microsatellite molecular markers for the Yangtze finless porpoise remains relatively limited, particularly concerning marker development and practical application. Although previous studies have employed microsatellite markers to assess genetic diversity populations from Poyang Lake and the Yangtze River, as well as to analyze kinship in ex situ populations (e.g., Tian’e-Zhou and Tongling) [11,12,13,14]. The majority of these markers were developed using traditional enrichment library screening and AFLP techniques [15,16,17,18]. These conventional methodologies exhibit notable technical limitations. For instance, the enriched library screening approach is a multi-stage procedure that involves the construction of a genomic library, hybridization-based screening, and sequence validation [19,20]. Additionally, the AFLP technique entails a series of complex procedures, including enzymatic digestion, ligation, and selective amplification, which necessitate a high level of technical proficiency [21,22]. These constraints have hindered its broader application in the field of conservation genetics. Notably, this study presents the first successful development of tetranucleotide microsatellite markers based on chromosome-level whole-genome data.

With the advancement of high-throughput sequencing technologies, high-quality chromosome-level genomic data have facilitated the efficient development of polymorphic microsatellite markers across the entire genome. A systematic genome-wide screening strategy not only significantly enhances development efficiency and reduces associated costs, but also enables the identification of highly polymorphic molecular markers [23]. In recent years, this strategy has proven highly effective in the development of microsatellite markers for various rare and endangered animal species. For example, Cai et al. [24] successfully identified 15 highly polymorphic and stable microsatellite loci by integrating the golden monkey (*Rhinopithecus roxellana*) genome with cross-population resequencing data. Huang et al. [25] developed 15 polymorphic markers based on the whole-genome sequence of the giant panda (*Ailuropoda melanoleuca*), thereby providing a novel genetic tool for analyzing its genetic relationships. In the selection of microsatellite markers, tetranucleotide repeat sequences are preferred due to their distinct advantages. Compared with dinucleotide or trinucleotide repeats, tetranucleotide microsatellites offer higher allelic resolution and reduced PCR amplification error rates [26,27]. Moreover, their fragment length polymorphism can be precisely detected using capillary electrophoresis, thereby minimizing genotyping errors caused by "stutter bands" (slippage artifacts) [28]. These characteristics make tetranucleotide microsatellite markers particularly suitable for applications such as population genetic analysis, kinship assessment, and related research domains.

To address the technical challenges associated with microsatellite marker development for the YFP, this study focuses on overcoming two key scientific challenges: (i) limitations of conventional methods, such as low efficiency and high costs; and (ii) the absence of a high-precision molecular marker development framework specifically suited to this species. By integrating chromosome-level high-quality genomic data with cross-population resequencing data, this study developed a set of tetranucleotide repeat microsatellite markers exhibiting stable amplification, high polymorphism, and low genotyping error rates. The application of this marker system to the genetic diversity analysis of the YFPs in Poyang Lake validated its reliability and effectiveness in population genetic studies. These markers not only serve as a critical molecular tool for understanding the genetic structure of the YFP and supporting the genetic management of ex situ conservation populations, but also as a methodological foundation for conservation genetics studies of other endangered aquatic mammal species.

## 2. Materials and Methods

### 2.1. Ethical Approval

The research related to the YFP has been approved by the relevant authorities. The experimental process strictly adheres to the national laws, regulations and policies concerning animal protection. It is compliant with the relevant provisions of the “Regulations on the Protection of Aquatic Wildlife in China” which were issued in 1993 and revised in 2013. The physical examinations conducted, such as animal chasing, handling and blood collection procedures, as well as related experiments, were approved by the Department of Agriculture and Rural Affairs of Jiangxi Province (Gan nong Zi (2022) 52 and 2022 [10]). Our study is reported in accordance with ARRIVE guidelines (https://arriveguidelines.org) (accessed on 19 August 2024).

### 2.2. Experimental Materials and Genome Resequencing

The 12 resequencing samples used to screen polymorphic microsatellite loci were collected during three distinct field operations: the 2021 relocation population health assessment in Jiajiang, Tongling; the 2023 relocation population examination in Xijiang, Anqing; and the 2022 emergency rescue operation conducted under extremely low water levels at Poyang Lake. The 15 samples of YFP used for the experimental validation of these polymorphic loci were collected in 2023 from the relocation group in Xijiang, Anqing, as well as from the deceased individuals in the Yangtze River main stream. The 50 samples used for genetic diversity analysis were collected during the emergency rescue operation under the extremely low water level at Poyang Lake in 2022–2023. Blood samples were all drawn from the tail veins of YFP using disposable sterile syringes and immediately stored in EDTA-K2 (Anticoagulant Bio-Tech Co., Ltd., Beijing, China). anticoagulant vacuum blood collection tubes. They were then transported back to the laboratory at −20 °C and stored at −80 °C in an ultra-low temperature refrigerator. Genomic DNA was extracted from their blood samples and whole-genome resequencing was then performed using the 2×150-bp paired-end mode of the DNBSEQ-T7 platform (MGI Tech, Shenzhen, China). Then, the raw reads were subjected to a series of quality control procedures to remove the low-quality reads. After stringent data filtering, each individual sample yielded an average of 22.4 Gb of clean data, with average mapping rate of 99.97%, sequencing depth of 25.44×, and coverage of 97.91% on the YFP reference genome. The clean reads were submitted to the NCBI Short Read Archive under BioProject accession number PRJNA1304169.

### 2.3. Polymorphic Microsatellite Locus Data Screening

This study identified a substantial set of microsatellite markers by integrating the high-quality chromosome-level genome of the YFP, previously published by the research team (PRJNA915046) [29] with the resequencing data from the aforementioned 12 samples. The screening process involved the following steps: First, the LobSTR reference index [30,31] was constructed based on the YFP genomic data using the lobstr_index.py script. Next, the resequencing data were aligned to the reference genome, and the resulting BAM files were sorted and processed using SAMtools (v1.9) [32]. From the alignment files generated from the 12 samples, allelic genotypes of microsatellite loci were identified. Finally, using VCFtools (v0.1.16) [33], tetranucleotide repeat microsatellite loci that were shared across all samples and contained more than three alleles were selected by applying the parameters “-min-alleles 3” and “-maf 0.1”.

### 2.4. Statistical Analysis of Microsatellite Loci Across Populations

Based on the shared microsatellite loci identified in the previous analysis, the Krait (1.5.1) software [34] was used to identify and quantify microsatellites commonly found in the genome of the YFP. A minimum repeat count of three was set as the threshold for tetranucleotide repeat motifs.

### 2.5. Experimental Validation of Polymorphic Microsatellite Loci

According to the principle of uniform distribution across chromosomes, 190 tetranucleotide microsatellite loci were randomly selected for experimental validation. A Perl script (v5.32.1) was used to extract 500-bp upstream and downstream flanking sequences surrounding the tetranucleotide repeat microsatellite loci from the whole-genome sequence. Primer design was carried out using Primer 5.0 software. The 5′ end of the forward primer F was linked to the M13 universal primer tag (5′-TGTAAAACGACGGCCAGT-3′). Primer synthesis was conducted by Shanghai Sangon Biotech Co., Ltd. (Shanghai, China).

Genomic DNA was extracted from 15 Yangtze finless porpoise samples using the QIAGEN DNeasy Blood & Tissue Kit (QIAGEN GmbH, Hilden, Germany) as the template. A total of 190 microsatellite loci was subsequently amplified via PCR. Each PCR reaction was carried out in a 25 μL reaction mixture consisting of 1 μL of DNA template, 0.5 μL each of forward and reverse primers, 0.5 μL of dNTP mix, 2.5 μL of 10× Taq buffer, 0.2 μL of Taq DNA polymerase, and ddH_2_O to reach the final volume. The PCR cycling conditions were as follows: initial pre-denaturation at 95 °C for 5 min; followed by 10 cycles of denaturation at 94 °C for 30 s, annealing at 60 °C for 30 s (with a decrement of 0.5 °C per cycle), and extension at 72 °C for 30 s; subsequently, 30 cycles of denaturation at 94 °C for 30 s, annealing at 55 °C for 30 s, and extension at 72 °C for 30 s; and a final extension at 72 °C for 10 min. Following purification of the PCR products, each samples was combined with HIDI loading buffer and the internal reference marker LIZ500 (both from ABI), and then analyzed by capillary electrophoresis using the 3730XL Genetic Analyzer (Applied Biosystems, Foster City, CA, USA). The amplified fragments were genotyped on the same platform to evaluate the efficacy and polymorphic characteristics of the primers.

Based on the results of preliminary PCR amplification and capillary electrophoresis, microsatellite loci with high amplification efficiency and significant polymorphism were selected for further optimization. The forward primers were individually labeled with fluorescent dyes (FAM, ROX, or HEX) at the 5′ ends. The fluorescently labeled primers were synthesized by Sangon Biotech (Shanghai) Co., Ltd. (Shanghai, China).and purified using high-performance liquid chromatography (HPLC) to ensure high purity. To maintain consistency, the PCR amplification and electrophoresis conditions used in the preliminary screening were applied in this stage. As a result, a set of high polymorphism and stable microsatellite markers was successfully developed.

### 2.6. Analysis of Genetic Diversity

Using the developed polymorphic microsatellite loci, a genetic diversity analysis was performed on a sample cohort of 50 Yangtze finless porpoise collected from Poyang Lake. Microsatellite polymorphism indices, such as the number of alleles (Na), observed heterozygosity (Ho), and expected heterozygosity (He), were calculated using GenAIEx 6.51 software [35]. Additionally, polymorphism information content (PIC) and null alleles frequency (Fnull) were analyzed using Cervus V3.0 software [36]. Linkage disequilibrium and Hardy-Weinberg equilibrium were assessed using Genepop 4.2 software [37].

## 3. Results

### 3.1. Screening and Validation of Four-Base Microsatellites Shared Across Populations

Based on the screening and comparison criteria described in the previous text, a total of 64,920 four-base microsatellite loci were identified as shared across 12 cross-group resequencing datasets. Statistical analysis revealed that the total length of these four-base microsatellites is 1,295,936 bp, accounting for 14.29% of all microsatellites identified across populations. The average length is 24.85 bp, with a relative abundance of 11.01 bp/Mb (Supplementary Table S1). The number of alleles ranges from 1 to 9, with 2, 3, and 4 alleles being the most frequently observed. The copy number of the repeat unit is mainly concentrated in the range of 5 to 19 times. The dominant repeat motif was (AAAT)n, which occurs 14,891 times and constitutes 28.55% of all repeat units.

Among the 64,920 shared four-base microsatellite loci identified across populations, a subset of 220 loci with more than three alleles was selected for further analysis. Of these, 190 loci were randomly selected for primer design, and specific primers were successfully developed for all targeted loci. To evaluate the applicability of these microsatellite markers, genomic DNA extracted from 15 samples of Yangtze finless porpoise was used as a template, and preliminary screening was performed using PCR amplification coupled with capillary electrophoresis. The results indicated that 149 primer pairs failed to produce effective amplification or displayed significant non-specific amplification when tested with DNA from field-dead samples. In contrast, 41 primer pairs exhibited robust amplification efficiency, minimal genotyping errors, and the associated microsatellite loci demonstrated satisfactory amplifiability and stability (Supplementary Table S2, Supplementary Figures S1–S4).

A set of fluorescently labeled primers was synthesized for the 41 four-base microsatellite loci identified during the initial screening phase. Subsequently, using the genomic DNA extracted from 15 Yangtze finless porpoise samples as templates, allele amplification and genotyping were carried out. The polymorphism of these 41 microsatellite loci was assessed using the allele frequency analysis module of Cervus 3.0 software. Finally, 19 highly polymorphic tetranucleotide repeat microsatellite loci, each containing 2 to 5 alleles, were selected (Table 1, the nucleotide sequences of the 19 loci are provided in Appendix A).

### 3.2. The Application of the Developed Polymorphic Microsatellite Markers

**Figure 1:**
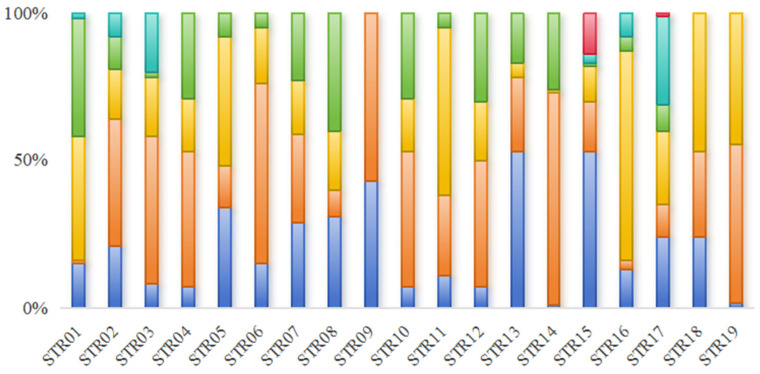
The microsatellite diversity distribution among the 50 Yangtze finless porpoise individuals sampled from Poyang Lake is illustrated in bar charts, where distinct colors denote different alleles at each microsatellite locus.

**Figure 2:**
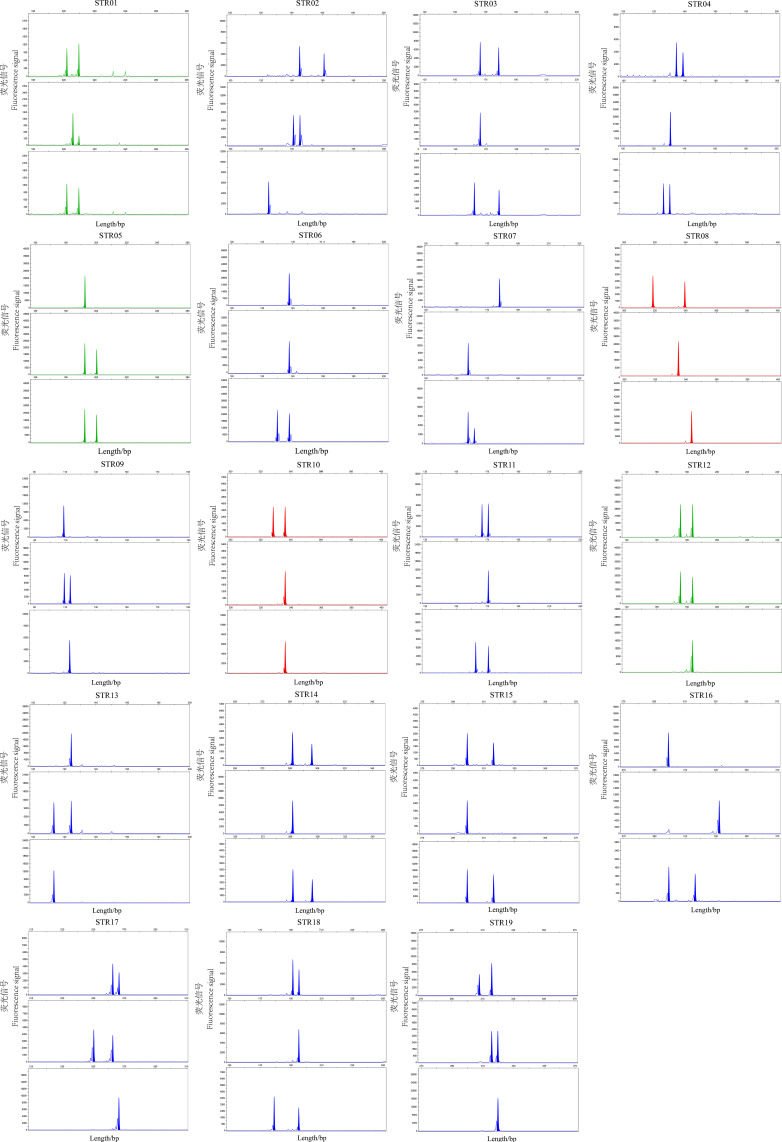
Genotyping results of 19 microsatellite loci in representative samples.

The genetic diversity of the YFPs in Poyang Lake was assessed using the developed microsatellite molecular markers (Supplementary Table S4). A total of 84 alleles (Na) were detected across 19 loci, with the number of alleles per locus ranging from 2 to 6 and an average of 4.42 (Figure 1). Observed heterozygosity (Ho) ranged from 0.44 to 0.84, with a mean value of 0.634; expected heterozygosity (He) ranged from 0.432 to 0.777, with a mean value of 0.649. The polymorphic information content (PIC) ranged from 0.370 to 0.732, with a mean value of 0.577 (Table 2). Except for STR09, STR14, and STR16, the remaining 16 loci (84%) exhibited high polymorphism, as indicated by PIC values greater than 0.5. Figure 2 displays the genotyping results of representative samples. Hardy-Weinberg equilibrium (HWE) analysis indicated that all 16 remaining loci are in HWE (*p* > 0.05), except for STR07, STR014, and STR16 (*p* < 0.05). Null allele detection revealed estimated F(null) values ranged from −0.280 to 0.359, with a mean of −0.015. Among these, four loci exhibited relatively high F(null) values (>0.05), suggesting a generally low frequency of null alleles. Furthermore, linkage disequilibrium analysis did not detect significant associations between any pair of loci (*p* < 0.01).

To evaluate and compare the detection efficiency of different microsatellite marker systems, this study analyzed the genetic diversity of ta YFP population in Poyang Lake using 20 microsatellite loci commonly reported in the literature [13]. A total of 78 alleles were detected across these loci, with the number of alleles per locus ranging from 2 to 6, and an average of 3.9. Observed heterozygosity (Ho) ranged between 0.177 and 0.900, with a mean value of 0.536, where as expected heterozygosity (He) ranged from 0.156 to 0.713, with an average of 0.562. The polymorphic information content (PIC) ranged between 0.15 and 0.691, with a mean value of 0.502 (Table 3). Among these, only 12 loci (60%) exhibited high polymorphism (PIC > 0.5). In contrast, the microsatellite marker system developed in this study demonstrated superior performance across all genetic diversity indices, particularly with a significantly higher proportion of highly polymorphic loci. These findings confirm that microsatellite markers developed through a whole-genome approach exhibit enhanced sensitivity in detecting genetic variations.

In summary, the microsatellite marker system developed in this study exhibits a high level of polymorphism (average PIC value of 0.577), strong genetic stability (84.2% of loci conforming to HWE), minimal interference of null alleles (average F(null) value of −0.001), and robust locus independence (absence of linkage disequilibrium).

## 4. Discussion

### 4.1. Development and Validation of Polymorphic Microsatellite Molecular Markers: Advantages and Assessment

This study used comparative analysis of genomic and cross-population resequencing data to identify a widely applicable tetranucleotide microsatellite marker. The method offers two key advantages. First, cross-population screening ensures consistent amplification across populations [38], strict allele-based selection criteria ensure high genetic informativeness [39]. This optimized screening approach has substantially improved the applicability and detection efficacy of microsatellite markers in population genetics [40]. Analysis of resequencing data from 12 YFP samples across four geographical populations identified 64,920 shared tetranucleotide microsatellite loci, providing a valuable resource for genetic studies across diverse populations.

Tetranucleotide microsatellites are preferred over traditional dinucleotide markers due to their greater amplification stability and a higher genotyping success rate in PCR [41,42]. For example, 21 of the 24 commonly used short tandem repeat (STR) loci on human autosomes (BioProject: PRJNA380127) are tetranucleotide markers, underscoring their practical value. This study revealed that tetranucleotide microsatellites in the YFP resequencing data are not only numerous but also notable polymorphic features. The key features are as follows: (i) 26 distinct motif types; (ii) a broad copy number range (4–40); (iii) high polymorphism across 12 resequencing samples. A total of 220 high-quality tetranucleotide microsatellite loci were identified using strict criteria (number of alleles > 3, PIC > 0.5). These loci not only confirm the applicability of tetranucleotide microsatellites in the YFP, but also provide valuable resources for developing an efficient microsatellite marker system.

Wild deceased samples of Yangtze River finless porpoises are often exposed to aquatic environments for long periods, making their DNA highly susceptible to degradation. This poses a significant challenge for downstream genetic analyses. To address this challenge, we evaluated 15 samples, including blood and field-collected deceased tissues, and screened 190 candidate microsatellite loci. Initially, 41 loci amplified successfully. After strict quality control, only 19 loci met all criteria: consistent amplification in degraded samples, low genotyping error rates, and high polymorphism levels (Supplementary Table S3). In this study, out of 190 candidate loci, 149 exhibited amplification failure. Following detailed analysis, the primary contributing factors were identified as follows: Firstly, DNA degradation resulting from prolonged exposure to aquatic environments may lead to the loss of the amplification template if the DNA break occurs within the primer binding region or a repetitive sequence. Secondly, the screening criteria stipulate that the initial amplification success rate must exceed 86%, which is higher than the 70% threshold documented in the literature, thereby excluding loci with suboptimal amplification efficiency [43]. The microsatellite markers identified in this study have been thoroughly validated and successfully address challenges in analyzing degraded samples. They provide reliable technical support for population genetic studies of the YFP. Notably, these markers remain stable under various sample preservation conditions, making them highly useful for field surveys and genetic monitoring of ex situ conservation populations of this species.

### 4.2. Assessment of the Efficacy of Microsatellite Molecular Markers and Their Implications for Population Genetics

This study used a comparative analytical approach to assess the effectiveness of the 19 newly developed microsatellite markers. A comparative analysis with the 20 microsatellite loci applied in prior studies of ex situ YFP conservation in Tian’e-Zhou [13] revealed that the marker system developed in this study exhibited notable advantages across multiple key genetic parameters. In this study, a total of 84 alleles were identified across the 19 loci (mean = 4.42), exceeding the 78 alleles reported in the earlier study (mean = 3.9), indicating greater sensitivity in detecting genetic variation. Furthermore, the average observed heterozygosity (Ho = 0.634) and expected heterozygosity (He = 0.649) were higher than those in the control study (Ho = 0.536, He = 0.562), showing improved ability to capture genetic diversity. Additionally, the average polymorphic information content (PIC = 0.577) was significantly higher than that in the control study (PIC = 0.502). Specifically, 16 loci (84.2%) exhibited high polymorphism (PIC > 0.5), compared to only 12 loci (60%) in the control study. These improvements likely resulted from stricter selection criteria during marker development, leading to better allele distribution and greater information content [44].

In the evaluation of microsatellite molecular marker, the Hardy-Weinberg equilibrium (HWE) test is a key indicator of marker validity. This study revealed that 3 of 19 microsatellite loci (STR07, STR14, and STR16) deviated significantly from Hardy-Weinberg equilibrium, suggesting potential biological relevance of endangered species. Several factors related to the Yangtze finless porpoise (YFP) population’s history may explain this deviation. First, habitat fragmentation in the middle and lower reaches of the Yangtze River may have led to hidden subpopulation structures within the Poyang Lake population [45,46,47]. Second, the YFP population has declined sharply from approximately 2700 individuals in 1991 to 1012 in 2017, may causing random changes in allele frequencies due to genetic drift [48,49]. Additionally, some microsatellite loci may be located in regions of the genome genomic affected by natural selection, such as those associated with immune response or environmental adaptation [50,51]. Notably, these deviated loci are not due to poor marker quality, but may instead offer valuable molecular insights into the population history and evolutionary dynamics of the YFP [52,53]. For example, significantly deviated loci may indicate genomic regions affected by selection or reflect genetic signatures of historical population events. Overall, the microsatellite marker system developed in this study improves polymorphisms detection and genetic diversity assessment while capturing key historical population traits. This marker system is suitable for analyzing genetic structure, assessing gene flow, and identifying conservation units in the YFP, ultimately offering solid genetic support for science-based conservation efforts.

## 5. Concluding Remarks and Future Perspectives

This study developed 19 polymorphic microsatellite markers for the YFP, showing high genetic diversity, broad applicability, and reliable performance. These markers are useful not only for assessing genetic diversity but also for analyzing genetic structure and identifying kinship, thereby supporting the scientific management of genetic resources and helping prevent inbreeding depression. Using whole-genome resequencing, the study also identified highly specific SNP loci in genes associated with adaptation, immune regulation, and disease resistance. This analysis reveals how SNP variations relate to environmental adaptability, improving our understanding of the population dynamics and environmental responses in the YFP. It provides a robust scientific foundation for species conservation, regulation of human activities, and habitat restoration.

## Figures and Tables

**Table 1:** Molecular marker sites of the Yangtze finless porpoise four-base repeat microsatellite and their corresponding primers.

Chromosome	Locus	Primer Sequences (5′-3′)	Fluorescent Labeling	Repeat Motif	Size Range
CH3	STR01	F:AAATATGCCTGTAGATGGATGGTT	5′HEX	(GATA)_12_	210–216
		R:AAGATGTTGAACTCCTTTATGCAAG			
CH4	STR02	F:AACTAGAAATACACACTGACACCAA	5′FAM	(AAAG)_10_	125–173
		R: TTTTCAGGAGAAATCCTTTTCTAC			
CH16	STR03	F: GCTGTCTCCTACCTTTTAATTATGA	5’FAM	(ATCT)_9_	163–179
		R:AAAGAAACACAGAAACATGCATTAT			
CH6	STR04	F: GCAGAGAAACAAAACCAAAAGG	5’FAM	(ATAG)_11_	127–139
		R: TCCTTAAAATCCATCTCTCCCTC			
CH7	STR05	F:AGAAAAACAGAACCAACAGGAGATA	5’HEX	(GATA)_9_	192–208
		R: ATAGTTGGTGGATGTTAATATTCCC			
CH13	STR06	F: TAAAAGAGACCAGACCATCTAGTCC	5’FAM	(AAGA)_11_	130–162
		R: TACATATTGGACCTAACAGATCAGC			
CH15	STR07	F: CTTGACAGATTGGAAATTAAAAACA	5’FAM	(CTAT)_5_	154–178
		R: TTTAATGCTAGCTGAAGTCGTTTC			
CH15	STR08	F: ATCAATCAGTCAGCTACCTATCCAC	5′ROX	(TCTA)_6_	320–344
		R: AGGGTCATGAACTTATAGAGCTTGT			
CH8	STR09	F: GTTGAATGTTGGCCTAAGTAATGA	5′FAM	(GGCA)_9_	109–113
		R: AAATCAGAGGCACATGAGACTTG			
CH3	STR10	F: CTACTGAGCCTGTTCTCTAGAGCC	5’ROX	(AAAG)_6_	328–344
		R: GGCAGAAAGAAATCTTACCTTTCTA			
CH13	STR11	F: GCTTTCTTGGTTTTACAGATTACAG	5′FAM	(TCTA)_10_	163–171
		R: CAGAGAAACAGAACCATTAGGAAA			
CH17	STR12	F: TCATTAACCTGCATAAGGGTCC	5′HEX	(TATG)_11_	176–184
		R: CATGCACCACAACGAAGAGTAG			
CH17	STR13	F: TGTGTGTATTAGTCAGTGTTCTCCA	5′FAM	(ATAG)_5_	113–133
		R: ATGTGAGCCAATTCTTGTAATAAAT			
CH2	STR14	F: CTCTGTGGATAAACAATGAACTGTG	5′FAM	(GAAG)_11_	291–303
		R: ACCCTGAGAAGAATTACCATTCTG			
CH2	STR15	F:TTCCTACATATTATGCACTACTGGG	5′FAM	(ATCT)_11_	300–332
		R:CTTAATGGAAAAGATTTCAGTTCCT			
CH10	STR16	F:TTAGGCACTGTTTTCAGTAGCATT	5′FAM	(TCTT)_13_	192–232
		R:GAGAAGTGAGTCAGGAGCCTTCTA			
CH10	STR17	F:CTGTGAAGGTACAGCATAAATGTGT	5′FAM	(TTTC)_10_	250–266
		R:CAGACTACTCCCTAAATGTAAAGCC			
CH14	STR18	F:GTAGAATCACAGACACTCAGAGTGG	5′FAM	(AGGT)_10_	179–195
		R:AATAATACTCCCAGATTTCTCCTCA			
CH10	STR19	F: GAACGCTCATTACCTAGGAACTTTA	5′FAM	(AAAC)_6_	308–320
		R: CATTTTCTGTCTGTAGGACTGAACA			

**Table 2:** Statistics of genetic parameters of 19 microsatellite molecular markers in 50 Yangtze finless porpoise samples.

Locus	Na	Ho	He	PIC	HWE	F(null)
STR01	5	0.82	0.647	0.569	NS	−0.128
STR02	5	0.62	0.730	0.684	NS	0.082
STR03	5	0.62	0.670	0.616	NS	0.019
STR04	4	0.68	0.685	0.609	NS	−0.009
STR05	4	0.62	0.672	0.604	NS	0.040
STR06	4	0.60	0.573	0.519	NS	−0.026
STR07	4	0.60	0.748	0.692	**	0.077
STR08	4	0.64	0.703	0.640	NS	0.042
STR09	2	0.54	0.647	0.370	NS	−0.048
STR10	6	0.84	0.685	0.627	NS	−0.118
STR11	4	0.44	0.594	0.529	NS	0.163
STR12	4	0.74	0.687	0.622	NS	−0.042
STR13	4	0.62	0.632	0.568	NS	0.008
STR14	4	0.42	0.414	0.343	**	−0.028
STR15	6	0.42	0.662	0.617	NS	0.198
STR16	5	0.42	0.474	0.442	*	−0.077
STR17	6	0.82	0.777	0.732	NS	−0.003
STR18	3	0.72	0.644	0.565	NS	−0.063
STR19	4	0.84	0.678	0.600	NS	−0.113
Mean	4.42	0.634	0.649	0.577		−0.001

Note: NS indicates no significant difference (*p* > 0.05); ** indicates very significant difference (*p* < 0.01), * indicates significant difference (*p* < 0.05).

**Table 3:** Statistical analysis of genetic parameters of 20 microsatellite loci in 50 samples of Yangtze finless porpoises.

Locus	Na	Ho	He	PIC	HWE	F(null)
NP404	3	0.477	0.481	0.375	NS	−0.028
Np409	6	0.367	0.694	0.629	***	0.182
Np428	3	0.177	0.156	0.150	NS	0.143
Np464	5	0.802	0.747	0.687	NS	−0.019
PPHO	4	0.700	0.739	0.691	*	0.154
YFP1	4	0.900	0.623	0.547	NS	−0.067
YFP8	3	0.367	0.465	0.381	NS	0.177
YFP42	4	0.633	0.629	0.553	NS	−0.142
YFP59	4	0.700	0.691	0.667	***	0.231
YFP69	3	0.500	0.495	0.441	NS	−0.128
SSR15	3	0.838	0.648	0.545	NS	0.163
SSR22	3	0.300	0.383	0.329	NS	−0.042
SSR40	3	0.565	0.584	0.519	NS	0.028
SSR41	4	0.511	0.583	0.505	NS	−0.048
SSR51	4	0.633	0.656	0.583	NS	−0.198
SSR63	3	0.468	0.575	0.489	NS	−0.177
SSR71	4	0.333	0.296	0.382	NS	−0.003
SSR73	4	0.602	0.663	0.614	NS	0.163
SSR5	6	0.403	0.713	0.654	***	−0.093
SSR75	6	0.453	0.422	0.315	NS	0.234
Mean	3.95	0.536	0.562	0.502		0.027

Note: NS indicates no significant difference (*p* > 0.05); *** Indicates a very significant difference (*p* < 0.001), * indicates significant difference (*p* < 0.05).

## Data Availability

Genome sequencing data have been deposited under the BioProject ID PRJNA1304169.

## Supplementary Materials

The following supporting information can be downloaded at: https://www.mdpi.com/article/10.3390/ani15172603/s1, Supplementary Figure S1. Genotyping outcomes of STR01–STR12 loci in representative sample sets. Supplementary Figure S2. Genotyping outcomes of STR13–STR24 loci in representative sample sets. Supplementary Figure S3. Genotyping outcomes of STR25–STR36 loci in representative sample sets. Supplementary Figure S4. Genotyping outcomes of STR37–STR41 loci in representative sample sets. Supplementary Table S1. Shared microsatellite loci types in the Yangtze finless porpoise. Supplementary Table S2. Preliminary screening of 41 pairs of primer information. Supplementary Table S3. Amplification performance of 41 microsatellite loci across 15 samples. Supplementary Table S4. Genotyping data for 50 samples collected from Poyang Lake across 19 microsatellite loci.

## Appendix A

Nucleotide Sequences of 19 Tetranucleotide Microsatellite Molecular Markers for the Yangtze Finless Porpoise

STR01:

AAATATGCCTGTAGATGGATGGTTCGATGGTTGGATGTTTGGATACATGGAAGGATGGATGGGAGGGAGGAAAGGATGGAGAGAGAGAATAGAT**GATAGATAGATAGATAGATAGATAGATAGATAGATAGATAGATAGATA**GATGAGTGAATCAATCAATTAAATAGATAAATGCCATACCTTACTTGCATAAAGGAGTTCAACATCTT;

STR02:

AACTAGAAATACACACTGACACCAATCTAGAACTGA**AAAGAAAGAAAGAAAGAAAGAAAGAAAGAAAGAAAGAAAG**AAAATTGCCCATCTTGAAACATGAACATTGAATCTTGATCCTGATTGGAGGTAGAAAAGGATTTCTCCTGAAAA;

STR03:

GCTGTCTCCTACCTTTTAATTATGATTATATAAATATAGATATAGAC**ATCTATCTATCTATCTATCTATCTATCTATCTATCT**AATCTCCTCTAAGGTACCTGTAAGGTGCATGAGCCCTGAGCCAACACTCCTTTTGGGGATAATGCATGTTTCTGTGTTTCTTT;

STR04:

GCAGAGAAACAAAACCAAAAGGATGTGT**ATAGATAGATAGATAGATAGATAGATAGATAGATAGATAGATAG**ATAAAGAGAGTCAGTGAAAGTAAGAGGGGGCGGTAGGGAGAGGGAGAGATGGATTTTAAGGA;

STR05:

AGAAAAACAGAACCAACAGGAGATAGGTGATAGATAAATAGATAGATAGGTAAGTAGATAGATGGATGGATG**GATAGATAGATAGATAGATAGATAGATAGATAGATA**GATGGATAATGAAACCTTTGAGCCAACCTGGGCTCTTTGGCTCTGTTGCTCTGGGCAACTCTGTGTATGTTGGGAATATTAACATCCACCAACTAT;

STR06:

TAAAAGAGACCAGACCATCTAGTCCAGAGGCTTAAAT**AAGAAAGAAAGAAAGAAAGAAAGAAAGAAAGAAAGAAAGAAAGA**AAAAAAAAGTTCTTTTTCCACCTCTGAACTTCTTTTTGATGGAAAGCTGATCTGTTAGGTCCAATATGTA;

STR07:

CTTGACAGATTGGAAATTAAAAACAAAATCAACTAGGCCTTCCCTACAGATCATTTCAGAATTATTACC**CTATCTATCTATCTATCTAT**CTCTATCTGTTCTGATTTTCTCTAGATCTTTTTCCTACGTGAGAAACGACTTCAGCTAGCATTAAA;

STR08:

ATCAATCAGTCAGCTACCTATCCACTCATCCATCTATTTATCATATTTATCATCTGTCATCTATCTATTTTATCTCCCTACCTTCTATATCTATCTCTCTCTTCTATCTCTCTCCCTTTATCTATCCATCATCTATCATCTGTCTGTCTATATCATCTATCATCTATTATCTATCTATCTATCATCTATCTATCTTCTATCTATCTATCTTCTATCTATCTATCATCTATCT**TCTATCTATCTATCTATCTATCTA**TCATCTATCATCTCATCTATCTCCCCCACTAGAATACAAGCTCTATAAGTTCATGACCCT;

STR09:

GTTGAATGTTGGCCTAAGTAATGACCAGAAATCTCACT**GGCAGGCAGGCAGGCAGGCAGGCAGGCAGGCAGGCA**GGCTGGTAGGCAGGCTTCCAAGTCTCATGTGCCTCTGATTT;

STR10:

CTACTGAGCCTGTTCTCTAGAGCCCGTGAGCCATAACTACCGAAGCTTATGCGCCTAGAGCCCATGCTCCGCAACAAGCCACCACAATAAGTAGCCCACGCACTGCAGTGAAGAGTAGCCCCTGTTTGCCGCAACTAGAGAAAGCCCGCATGCAGCAACGAAGACCCAACACAGCCAAAAAT**AAAGAAAGAAAGAAAGAAAGAAAGAAAAG**AAAACAAAAAGGGTCAACTGTACTTTTACAGAAGAATAAATTATCATTTATATTCAGAACATGTATGTAACTGATATATCTGCCTGGTTATATAGAAAGGTAAGATTTCTTTCTGCC;

STR11:

GCTTTCTTGGTTTTACAGATTACAGATTGCAAATTGTGATACTTCTCAACCTCATAATTGTGTGAGTCAATTCCTATAATAAATC**TCTATCTATCTATCTATCTATCTATCTATCTATCTATCTA**TCATCTCTCCTTATATATTTCCTAATGGTTCTGTTTCTCTG;

STR12:

TCATTAACCTGCATAAGGGTCCTGTGATGTAGGGATTAACCTCCCAATTT**TATGTATGTATGTATGTATGTATGTATGTATGTATGTATGTATG**GCTGCACTGGGTCCTCGTTGCTGTGCACGAGCTTTCTCTAGTTGCGGCGAGTGGGGGCTACTCTTCGTTGTGGTGCATG;

STR13:

TGTGTGTATTAGTCAGTGTTCTCCAGAGAAACAGAACCAATAGGAAATTAGATA**ATAGATAGATAGATAGATAG**GTAGATAGATAGATTAGATTTATTACAAGAATTGGCTCACAT;

STR14:

CTCTGTGGATAAACAATGAACTGTGAAAAAAAACAATTAACTGTACTCTAAAATAGGTGATTTATATATGTGAAATATATCCCAGTAAAGCTGAGGAAAGAAGGAGGGAGGAAGGGAAGGAGGGAAGGAAGGAAAGAAGGAAGGAAGGAAGGACA**GAAGGAAGGAAGGAAGGAAGGAAGGAAGGAAGGAAGGAAGGAAG**GAGGCAGGGAGGAAGGGAGGGAAGAAGGAAGGAATGAAAGATACAGATCTGAAGTGAAGTGTCTCACCCAGAATGGTAATTCTTCTCAGGGT;

STR15:

TTCCTACATATTATGCACTACTGGGACAGTTCCTTCCTTCCTTCCATCTATC**ATCTATCTATCTATCTATCTATCTATCTATCTATCTATCTATCT**ATCAACTGGTTGGCCAAAAGTGCCTTCACTTTTTAAGTAAAAATAAAAGACACATTTTTCATTTTCACCAAGAACTTTATTGGACAACGTATTCACCCTTTTGTCCCACTACCATCTGCCGTTTTTCAGGCAACTTCATGATTCCATCTTCCCAAAACTTTTTATCTTTTTGAGCAAAGAACTGTTCCGGGTGCCTTTTACAGTTTTCCAAGGAACTGAAATCTTTTCCATTAAG;

STR16:

TTAGGCACTGTTTTCAGTAGCATTGCTATGGATAGTAATGTTTTCTTTCTTTCTCTCTCTCTTTCTC**TCTTTCTTTCTTTCTTTCTTTCTTTCTTTCTTTCTTTCTTTCTTTCTTTCTT**ATCTTCATCTTCCAGTTTTTCTAATTATTTCATATGAAATGAATAATCCCATGGTCCAATCCCCAGTCATCCATAGAAGGCTCCTGACTCACTTCTC;

STR17:

CTGTGAAGGTACAGCATAAATGTGTGAAAATAAAATATCTCCTGACCTCGCCATCAGAGACATAGATTTTATTTTTTTCTTAGGAGTGTCTGGTGAGTTTTCTTTCTTTCTTTCCTTTCTTTCTCTTAT**TTTCTTTCTTTCTTTCTTTCTTTCTTTCTTTCTTTCTTTC**CTCTCTCTCTCTCTCTTTCTTTCTTCTTCTCCCTTTTTTTTGGTGGTGGTTGTCGCAGCTCAACGGGGCTTTACATTTAGGGAGTAGTCTG;

STR18:

GTAGAATCACAGACACTCAGAGTGGTGAGGGCAGGAACTCTGCCAGGGAGCCCTGGGCCGGGGCAAGGATGTCCTTCCTGCTACCACCACAGGGAGGG**AGGTAGGTAGGTAGGTAGGTAGGTAGGTAGGTAGGTAGGT**AGCTGGCTGTCTCCTCTCATATCCTTGGCACATGAGGAGAAATCTGGGAGTATTATT;

STR19:

GAACGCTCATTACCTAGGAACTTTAATTAATCTAGTTGAACTTAAGCTTAAAGAAATAATTGTTGGGGCTTCCCTGGTGGCGCAGTGGTTGGGAGTCCGCCTGCCGATGCAGGGGACACGGGTTCGTGCCCCGGTCTGGGAGGATCCCACATGCCGCGGAGCGGCTGGGCTCGTGAGCCATGGCCGCTGAGCCTGCGCGTCCGGAGCCTGTGCTCCACAACGGGAGAGGTCACAACAGTGAGAGGCCCGCGTACCGCAAA**AAACAAACAAACAAACAAACAAAC**AAAAAAAAAAAGAAATAATTGTTCAGTCCTACAGACAGAAAATG

## References

[1] Gao A.L., Zhou K.Y. (1995). China academic journal electronic publishing house. Acta Theriol. Sin..

[2] Mei Z.G., Hao Y.J., Zheng J.S., Wang Z.T., Wang K.X., Wang D. (2021). Population status and conservation outlooks of the Yangtze Finless Porpoise in the Lake Poyang. J. Lack Sci..

[3] Min J.L., Que J.L., Tian Z., Rao R.C. (2023). Habitat analysis of yangtze finless porpoises in Po-yang Lake under Extremely Low Water Level. Acta Agric. Univ. Jiangxiensis.

[4] Wang D., Turvey S., Zhao X., Mei Z. (2013). Neophocaena asiaeorientalis ssp. asiaeorientalis. The IUCN Red List of Threatened Species, Version 3.

[5] Yuan Y., Zhang P.J., Wang K., Liu M.Z., Li J., Zheng J.S., Wang D., Xu W.J., Lin M.L., Dong L.J. (2018). Genome Sequence of the Freshwater Yangtze Finless Porpoise. Genes.

[6] Xu P., Liu K., Ying C.P., Zhang J.L. (2024). Progress and prospects on the protection of Yangtze Finless Porpoise. Acta Hydrobiol. Sinica.

[7] Chen B.Y., Xin Y., Lu F.T., Sun J., Liu S., Li M., Wu B., Wang C.R. (2023). Monitoring Status and Prospects of Yangtze Finless Porpoise. Environ. Monit. China.

[8] Cavagnaro P.F., Senalik D.A., Yang L. (2010). Genome wide characterization of simple sequence repeats in cucumber (*Cucumis sativus*). BMC Genom..

[9] Dai X.G., Fang Z.X., Hu X.C., Han Q.Z., Guo R.R., Gu R. (2021). Whole genome SSR analysis and polymorphic primer screening in *Carya illinoinensis*. Mol. Plant Breed..

[10] Chen H.L. (2022). Population Genetics of *Paramisgurnus dabryanus* in China Based on Mtana and Microsatellite Markers. Master’s Thesis.

[11] Wang R.Y., Chen M.M., Wan X.L., Tang B., Hao Y.J., Fan F., Wang K.X. (2023). Microsatellite genetic diversity evaluation and development prediction of the Yangtze finless porpoises population the Poyang Lake. Acta Hydrobiol. Sin..

[12] Zhang F., Zhang B.W., Tang W.Q., Liu J., Wu J.H., Tang B. (2018). Analysis of genetic diversity and population dynamics of the narrow-ridged finless porpoise in the Yangtze River Estuary. J. Shanghai Ocean. Univ..

[13] Chen M.M., Zheng J.S., Gong C., Zhao Q.Z., Wang D. (2014). Inbreeding evaluation on the Ex Situ conserved Yangtze Finless Porpoise population in Tian’ezhou national natural reserve. Chin. J. Zool..

[14] Feng J.W., Zheng J.S., Zhou Z., Lin G., Wang D., Zheng B.Y. (2009). Paternity determination of Captivity-Bred Yangtze Finless Porpoises *Neophocaena phocaenoides asiaeorientalis* by microsatellite genotyping. Prog. Mod. Biomed..

[15] Chen L., Yang G. (2008). Development of tetranucleotide microsatellite loci for the finless porpoise (*Neophocaena phocaenoides*). Conserv. Genet..

[16] Chen L., Bruford M., Yang G. (2007). Isolation and characterization of microsatellite loci in the finless porpoise (*Neophocaena phocaenoides*). Mol. Ecol. Notes.

[17] Zhou Z., Zheng J.S., Chen M.M., Zhao Q.Z., Wang D. (2012). Genetic evaluation and development prognosis on ex situ conserved Yangtze finless porpoises living in Tian’e Zhou national natural reserve. Acta Hydrobiol. Sin..

[18] Zheng J.S., Liao X.L., Tong J.G. (2008). Development and characterization of polymorphic microsatellite loci in the endangered Yangtze finless porpoise (*Neophocaena phocaenoides asiaeorientalis*). Conserv. Genet..

[19] Zhang Y., Lou F.R., Han Z.Q. (2019). Development of microsatellite markers in oratosquilla oratoria Transcriptome. J. Zhejiang Ocean Univ..

[20] Han C.H. (2016). Development and Application of Microsatellite DNA Markers for The Black Rockfish. *Sebastes schlegelii*. Master’s Thesis.

[21] Guo Y.W., Sun K.X., Tian C., Cao T., Zhao H.D., Sun X.Z. (2018). Application status analysis of molecular marker in sheep breeding. China Anim. Husb..

[22] Xia X.R., Kuang H.Y., Sun D.P., Lu Y., Zheng H.J., Hu Y.X. (2025). Analysis of genetic diversity and population genetic structure of sweet maize inbred lines using SNP markers. Acta Agric. Shanghai.

[23] Yang B., Lin L., Li C.H., Xu S.N., Liu Y., Xiao Y.Y., Chen Z.Z. (2015). Development and evaluation of microsatellite markers in Parargyrops edita. South China Fish. Sci..

[24] Cai Y.S., Yu H.Y., Liu H., Jiang C., Sun L., Niu L.L., Liu X.Z., Li D.Y., Li J. (2020). Genome-wide screening of microsatellites in golden snub-nosed monkey (*Rhinopithecus roxellana*), for the development of a standardized genetic marker system. Sci. Rep..

[25] Huang J., Li Y.Z., Du L.M., Yang B., Shen F.J., Zhang H.M., Zhang Z.H., Zhang X.Y., Yue B.S. (2015). Genome-wide survey and analysis of microsatellites in giant panda (*Ailuropoda melanoleuca*),with a focus on the applications of a novel microsatellite marker syste. BMC Genom..

[26] Walsh P.S., Fildes N.J., Reynolds R. (1996). Sequence analysis and characterization of stutter products at the tetranucleotide repeat locus vWA. Nucleic Acids Res..

[27] Perinchery G., Nojima D., Goharderakhshan R., Tanaka Y., Dahiya R. (2000). Microsatellite instability of dinucleotide tandem repeat sequences is higher than trinucleotide, tetranucleotide and pentanucleotide repeat sequences in prostate cance. Int. J. Oncol..

[28] Han X.K., Li J.L., Wang Z.Q., Bai Z.Y. (2014). Development and characteristics of tetranucleotide repeat microsatellite loci in *Hyriopsis cumingii*. Biotechnol. Bull..

[29] Yin D.H., Chen C.H., Lin D.Q., Zhang J.L., Cao Z.C., Zhang H. (2024). Telomere-to-telomere gap-free genome assembly of the endangered Yangtze finless porpoise and East Asian finless porpoise. Gigascience.

[30] Gymrek M., Golan D., Rosset S., Erlich Y. (2012). LobSTR: A short tandem repeat profiler for personal genomes. Genome Res..

[31] Zhou X.M., Meng X.H., Liu Z.J., Chang J., Wang B.S., Li M.Z. (2016). Population genomics reveals low genetic diversity and adaptation to hypoxia in snub-nosed monkeys. Mol. Biol. Evol..

[32] Li H., Handsaker B., Wysoker A., Fennell T., Ruan J., Marth G. (2009). The Sequence alignment/map(SAM)format and SAMtools. Bioinformatics.

[33] Danecek P., Auton A., Abecasis G., Albers C.A., Banks E., DePristo M.A., Handsaker R.E., Lunter G., Marth G.T., McVean G. (2011). The variant call format and VCFtools. Bioinformatics.

[34] Du L.M., Zhang C., Liu Q., Zhang X.Y., Yue B.S. (2017). Krait: An ultrafast tool for genome-wide survey of microsatellites and primer design. Bioinformatics.

[35] Peakall R., Smouse P.E. (2012). GenAIEx 6.5: Genetic analysis in Excel. Population genetic software for teaching and research-an update. Bioinformatics.

[36] Kalinowski S.T., Taper M.L., Marshall T.C. (2007). Revising how the computer program CERVUS accommodates genotyping error increases success in paternity assignment. Mol. Ecol..

[37] Rousset F. (2008). Genepop’007: A complete re-implementation of the Genepop software for Windows and Linux. Mol. Ecol. Resour..

[38] Amino O., Badaoui B., Machmoun M., Piro M. (2024). Evaluation of the effectiveness of single nucleotide polymorphisms compared to microsatellite markers for parentage verification in Moroccan horse. Anim. Genet..

[39] Chapuis M.P., Estoup A. (2007). Microsatellite null alleles and estimation of population differentiation. Mol. Biol. Evol..

[40] Du Y.Y., Yang Z.Y. (2025). Microsatellite loci isolation for three schizothoracine species and their applications on analysis of genetic polymorphism. Chin. J. Zool..

[41] Archie E.A., Moss C.J., Alberts S.C. (2003). Characterization of tetranucleotide microsatellite loci in the African Savannah Elephant (*Loxodonta africana africana*). Mol. Ecol..

[42] Fernando P., Vidya T.N.C., Melnick D.J. (2001). Isolation and characterization of tri-and tetranucleotide microsatellite loci in the Asian elephant, Elephas maximus. Mol. Ecol..

[43] Cai Y.S. (2021). Establishment and Optimization of Microsatellite Marker System for Golden Snub-Nosed Monkey, and Analysis of the Methylation Profiles of Two Tissues. Ph.D. Thesis.

[44] Selkoe K.A., Toonen R.J. (2006). Microsatellites for ecologists: A practical guide to using and evaluating microsatellite markers. Ecol. Lett..

[45] Zhao X., Wang D., Turvey S.T., Taylor B., Akamatsu T. (2013). Distribution patterns of Yangtze finless porpoises in the Yangtze River: Implications for reserve management. Anim. Conserv..

[46] Wang C.R., Suo W.W., Jiang G.M., Li H.M., Liang Z.Q., Yang X., Yuan X.P., Li H., Liao F.C., Ge H.Z. (2019). Spatial distribution of the Yangtze finless porpoise and relationship to fish density in East Dongting Lake, China. China Environ. Sci..

[47] Zhang H., Yin D.H., Que J.L., Zhu X.Y., Lin D.Q., Ying C.P., Yu J.X., Liu K. (2025). Genetic diversity and population differentiation of Yangtze Finless Porpoise in Poyang Lake. Animals.

[48] Spencer C.C., Neigel J.E., Leberg P.L. (2000). Experimental evaluation of the usefulness of microsatellite DNA for detecting demographic bottlenecks. Mol. Ecol..

[49] Li Y.X., Shao C.L., Gao H.R., Wu J.S., Xu M.Q., Qang Y.P., Liu H.J., Su J.Y., Shan W.J. (2025). Genetic diversity and structure analysis of the *Equus hemionus* hemionus in Kalamaili. Chin. J. Anim. Vet. Sci..

[50] Yang W.J. (2023). Study on Genetic Diversity and Whole Genome Selection Signals of Hainan Cattle Population. Master’s Thesis.

[51] Zhou T. (2023). Genetic Differentiation and Genetic Diversity of Rhesus Macaques in Sichuan Base on Microsatellite Markers. Master’s Thesis.

[52] Johnson M.S., Black R. (1984). The Wahlund effect and the geographical scale of variation in the intertidal limpet *Siphonaria* sp. Mar. Biol..

[53] Lotterhos K.E. (2015). Whitlock, MC. The relative power of genome scans to detect local adaptation depends on sampling design and statistical method. Mol. Ecol..

