# Supplementary material for: Development and Validation of Tetranucleotide Repeat Microsatellite Markers at the Whole-Genome Level in the Yangtze Finless Porpoise
Source: Animals (Basel). 2025 Sep 4;15(17):2603. doi: 10.3390/ani15172603 (PMC12427282; doi:10.3390/ani15172603)
Supplement: Supplementary file 1 [file animals-15-02603-s001.zip › Supplementary Materials.pdf]

Supplementary Materials

Supplementary Figure S1 Genotyping outcomes of STR01–STR12 loci in repr esentative sample sets.

Supplementary Figure S2 Genotyping outcomes of STR13–STR24 loci in repr esentative sample sets.

Supplementary Figure S3 Genotyping outcomes of STR25–STR36 loci in repr esentative sample sets.

Supplementary Figure S4 Genotyping outcomes of STR37–STR41 loci in repr esentative sample sets.

Supplementary Table S1 Shared microsatellite loci types in the Yangtze finles s porpoise.

Supplementary Table S2 Preliminary screening of 41 pairs of primer information.

Supplementary Table S3 Amplification performance of 41 microsatellite loci across 15 samples.

Appendix Nucleotide Sequences of 19 Tetranucleotide Microsatellite Molecular Markers for the Yangtze Finless Porpoise

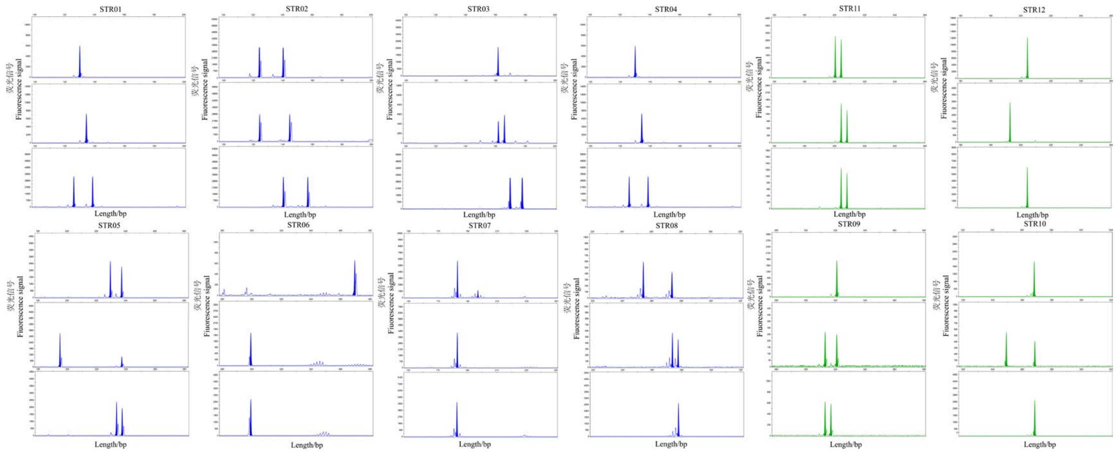

Supplementary Figure S1 Genotyping outcomes of STR01 – STR12 loci in representative sample sets.

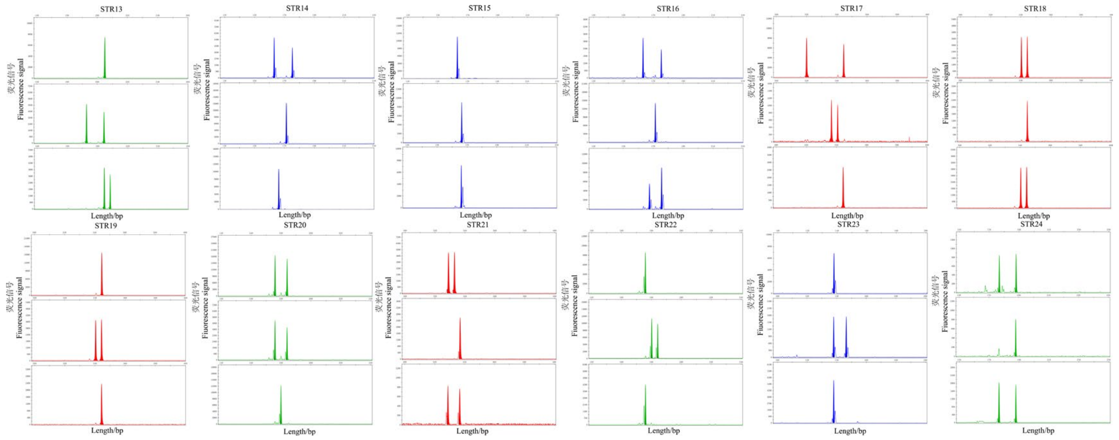

Supplementary Figure S2 Genotyping outcomes of STR13 – STR24 loci in representative sample sets.

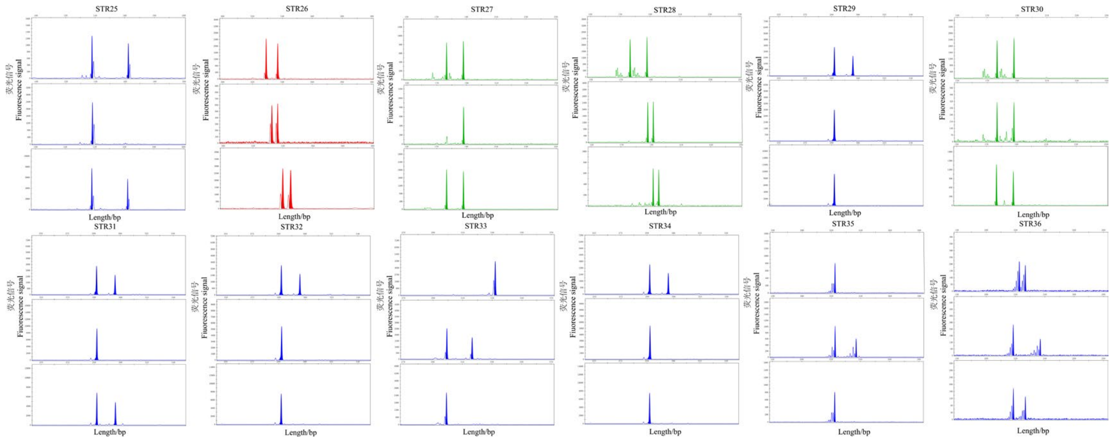

Supplementary Figure S3 Genotyping outcomes of STR25 – STR36 loci in representative sample sets.

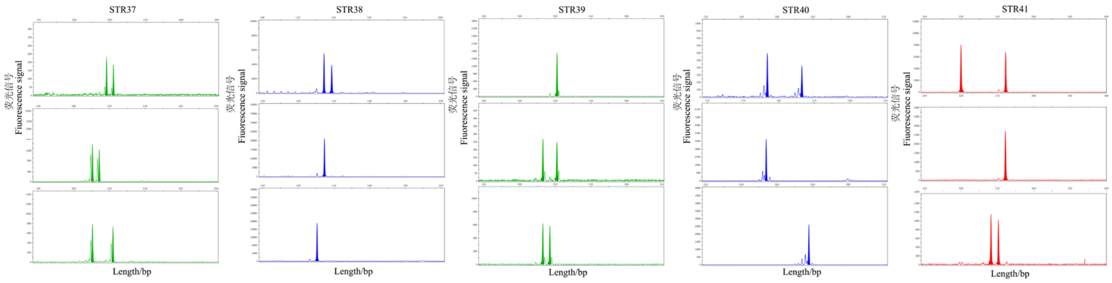

Supplementary Figure 4 Genotyping outcomes of STR37–STR41 loci in representative sample sets.

Supplementary Table S1 Shared microsatellite loci types in the Yangtze finless porpoise.

Repeat type	Total Number	Length/bp	Proportion/%	Average length/bp	Relative abundance/(loci/Mb)
Dinucleotide	307324	5781582	67.62	21.75	45.15
Trinucleotide	46799	527445	10.29	17.3	6.91
Tetranucleotide	64920	1295936	14.29	24.85	11.01
Pentanucleotide	4993	137425	1.09	32.65	0.81
Hexanucleotide	30408	1250454	6.69	37.85	9.12

Supplementary Table S2 Preliminary screening of 41 pairs of primer information.

Chromosome	Locus	Primer sequences	Fluorescent labeling	Repeat motif	Size range
CH3	STR01	F:AAATATGCCTGTAGATGGATGGTT<br>R:AAGATGTTGAACTCCTTTATGCAAG	5'HEX	$(GATA)_{12}$	202-210
CH4	STR02	F:AACTAGAAATACACACTGACACCAA<br>R: TTTTCAGGAGAAATCCTTTTCTAC	5'FAM	$(AAAG)_{10}$	125-161
CH16	STR03	F: GCTGTCTCCTACCTTTTAATTATGA<br>R:AAAGAAACACAGAAACATGCATTAT	5'FAM	$(ATCT)_{9}$	163-171
CH6	STR04	F: GCAGAGAAACAAAACCAAAAGG<br>R: TCCTTAAAATCCATCTCTCCCTC	5'FAM	$(ATAG)_{11}$	127-139
CH7	STR05	F:AGAAAAACAGAACCAACAGGAGATA<br>R: ATAGTTGGTGGATGTTAATATTCCC	5'HEX	$(GATA)_{9}$	192-204
CH13	STR06	F: TAAAAGAGACCAGACCATCTAGTCC	5'FAM	$(AAGA)_{11}$	130-146

		R: TACATATTGGACCTAACAGATCAGC			
CH15	STR07	F: CTTGACAGATTGGAAATTAAAAACA<br>R: TTTAATGCTAGCTGAAGTCGTTTC	5'FAM	$(CTAT)_5$	154-178
CH15	STR08	F: ATCAATCAGTCAGCTACCTATCCAC<br>R: AGGGTCATGAACTTATAGAGCTTGT	5'ROX	$(TCTA)_6$	320-344
CH8	STR09	F: GTTGAATGTTGGCCTAAGTAATGA<br>R: AAATCAGAGGCACATGAGACTTG	5'FAM	$(GGCA)_9$	109-113
CH3	STR10	F: CTACTGAGCCTGTTCTCTAGAGCC<br>R: GGCAGAAAGAAATCTTACCTTTCTA	5'ROX	$(AAAG)_6$	328-336
CH13	STR11	F: GCTTTCTTGGTTTTACAGATTACAG<br>R: CAGAGAAACAGAACCATTAGGAAA	5'FAM	$(TCTA)_{10}$	167-175
CH17	STR12	F: TCATTAACCTGCATAAGGGTCC<br>R: CATGCACCACAACGAAGAGTAG	5'HEX	$(TATG)_{11}$	176-184
CH17	STR13	F: TGTGTGTATTAGTCAGTGTTCTCCA<br>R: ATGTGAGCCAATTCTTGTAATAAAT	5'FAM	$(ATAG)_5$	113-133
CH2	STR14	F: CTCTGTGGATAAACAATGAACTGTG<br>R: ACCCTGAGAAGAATTACCATTCTG	5'FAM	$(GAAG)_{11}$	291-303
CH2	STR15	F:TTCCTACATATTATGCACTACTGGG<br>R:CTTAATGGAAAAGATTTCAGTTCCT	5'FAM	$(ATCT)_{11}$	316-332
CH10	STR16	F:TTAGGCACTGTTTTCAGTAGCATT<br>R:GAGAAGTGAGTCAGGAGCCTTCTA	5'FAM	$(TCTT)_{13}$	216-232
CH10	STR17	F:CTGTGAAGGTACAGCATAAATGTGT<br>R:CAGACTACTCCCTAAATGTAAAGCC	5'FAM	$(TTTC)_{10}$	250-262
CH14	STR18	F:GTAGAATCACAGACACTCAGAGTGG<br>R:AATAATACTCCCAGATTTCTCCTCA	5'FAM	$(AGGT)_{10}$	179-195
CH10	STR19	F: GAACGCTCATTACCTAGGAACTTTA<br>R: CATTTTCTGTCTGTAGGACTGAACA	5'FAM	$(AAAC)_6$	308-320
CH1	STR20	F: ATCATTAGTCTTTGTAGTACCAGGG<br>R: GTATCATATCCTCCTTATCCGTCT	5'HEX	$(AAGG)_7$	193-213

CH3	STR21	F: TGGAAAGTATTTGGACAATAGGTAA<br>R: GTTTCTCTTCGTCTCTCCTTCAG	5'ROX	$(TTTG)_{12}$	247-267
CH18	STR22	F: CAGAAGTGCATGAGAGACAGAATAG<br>R: AAATAAGTTTACAGAAGGAGCATCG	5'FAM	$(CCCA)_{22}$	108-128
CH2	STR23	F:AGAACTATAGGTTCTTTGCCTCTTG<br>R:CCTTTGGTATTTATCTGGAGAGTGA	5'FAM	$(AAAC)_{11}$	131-139
CH6	STR24	F:ATCCTGCTAACATCTAATATTCCAA<br>R:TTGCTTCTTTTAAATAGCAAGTGTT	5'HEX	$(ATATA)_{6}$	211-226
CH7	STR25	F:ATAATCTCTCTCCATAAACACATGC<br>R:ACAGGAATCTTAACCATGTAATGTG	5'HEX	$(TCTA)_{11}$	203-243
CH11	STR26	F:GAAGAAACCAAGTCCAAAACTAAAG<br>R: ACAGTATTGGACTGTGAACAAGTGA	5'ROX	$(TCTA)_{6}$	266-278
CH3	STR27	F:CACATTTTTATCCGTTTTATTCACT<br>R:TAATTATGATACAAAGGGAATTTGG	5'HEX	$(TCTA)_{6}$	213-225
CH9	STR28	F:AACTACTGAGCCCATGTGCCTA<br>R:GCCTCTTCCATTTTCTGTGACTC	5'ROX	(AATT)7	246-286
CH11	STR29	F:AGGATCTGAAAGCAGCTCATTAT<br>R:GCATTAGCAGGCAGATTGTCA	5'ROX	$(AAAG)_{6}$	231-239
CH11	STR30	F:CAATGCAGGGGATGCAGGTT<br>R:TTTTCTCAGGGTTTACGCCCA	5'ROX	$(TCTA)_{10}$	254-258
CH13	STR31	F:TCAATTATCCTTATCCCCACTTG<br>R:CTGTACATTTAGACCCAGCCCT	5'ROX	$(TATG)_{11}$	250-270
CH9	STR32	F:TTTTAAATCACCAAAGATTATTTGG<br>R:ATTTCCAAACAGACAACTGAGGT	5'ROX	$(ATAG)_{5}$	281-285
CH15	STR33	F:TCCTCTTATTTTTCTATTGTCCCTT<br>R:TTTGATGATGATTACCTTCCTTTTA	5'FAM	$(GAAG)_{11}$	176-180
CH8	STR34	F:CACACTCACCTATCCATCAACCA<br>R:CTGATCATTTTCTAGGTCCCTGG	5'ROX	$(ATCT)_{11}$	469-489
CH16	STR35	F:TCAAGAAGAGTGCCAAAACATAAG	5'FAM	$(TCTT)_{13}$	204-224

		R;CAATGAAAAGCTTTGTCTTCTCTAG			
CH2	STR36	F:GCAATGAGACACAAGTAAGAACAAA<br>R:TGGAACTCTATTGATCACTGAAATC	5'ROX	$(TTTC)_{10}$	221-225
CH18	STR37	F:TGATTCACTTTGTTATACAGCAGAA<br>R:CCTCTCCAGCATTTATTATTCATAG	5'HEX	$(AGGT)_{10}$	174-198
CH4	STR38	F: CTTGTTTACAATGGACCGTGC<br>R: CAGACCTAAGGGAGAGCTCCA	5'FAM	$(TATT)_{8}$	151-171
CH15	STR39	F:CTGGGGTATATGTATTTGCAAGAG<br>R:TGAAATGAATGAATCCTAATCTCAA	5'FAM	$(TATC)_{12}$	168-172
CH18	STR40	F:TAATCATGTTAACCCTATCTTCTGG<br>R:GAACTAAGATCCTACAAGCCACAT	5'FAM	$(TATT)_{7}$	102-114
CH19	STR41	F:ATGATATTTAAGAGATGCTATGGGA<br>R:GGTTAATCTCCTACCTACTGCAGTT	5'HEX	$(TATC)_{12}$	195-207

Supplementary Table S3 Amplification performance of 41 microsatellite loci across 15 samples.

Locus	Primer sequences(5′-3′)	Amplification success rate	Repeat motif	N	Size range	Genotyping success rat
STR01	F:AAATATGCCTGTAGATGGATGGTT<br>R:AAGATGTTGAACTCCTTTATGCAAG	100%	$(GATA)_{12}$	3	202-210	100%
STR02	F:AACTAGAAATACACACTGACACCAA<br>R: TTTTCAGGAGAAATCCTTTTCTAC	86.7%	$(AAAG)_{10}$	5	125-161	100%
STR03	F: GCTGTCTCCTACCTTTTAATTATGA<br>R:AAAGAAACACAGAAACATGCATTAT	100%	$(ATCT)_{9}$	3	163-171	100%
STR04	F: GCAGAGAAACAAAACCAAAAGG<br>R: TCCTTAAAATCCATCTCTCCCTC	93.3%	$(ATAG)_{11}$	3	127-139	100%
STR05	F:AGAAAAACAGAACCAACAGGAGATA<br>R: ATAGTTGGTGGATGTTAATATTCCC	100%	$(GATA)_{9}$	3	192-204	100%
STR06	F: TAAAAGAGACCAGACCATCTAGTCC<br>R: TACATATTGGACCTAACAGATCAGC	93.3%	$(AAGA)_{11}$	5	130-146	100%

STR07	F: CTTGACAGATTGGAAATTAAAAACA<br>R: TTTAATGCTAGCTGAAGTCGTTTC	100%	$(CTAT)_5$	4	154-178	100%
STR08	F: ATCAATCAGTCAGCTACCTATCCAC<br>R: AGGGTCATGAACTTATAGAGCTTGT	100%	$(TCTA)_6$	3	320-344	100%
STR09	F: GTTGAATGTTGGCCTAAGTAATGA<br>R: AAATCAGAGGCACATGAGACTTG	100%	$(GGCA)_9$	2	109-113	100%
STR10	F: CTACTGAGCCTGTTCTCTAGAGCC<br>R: GGCAGAAAGAAATCTTACCTTTCTA	93.3%	$(AAAG)_6$	3	328-336	100%
STR11	F: GCTTTCTTGGTTTTACAGATTACAG<br>R: CAGAGAAACAGAACCATTAGGAAA	100%	$(TCTA)_{10}$	3	167-175	100%
STR12	F: TCATTAACCTGCATAAGGGTCC<br>R: CATGCACCACAACGAAGAGTAG	100%	$(TATG)_{11}$	3	176-184	100%
STR13	F: TGTGTGTATTAGTCAGTGTTCTCCA<br>R: ATGTGAGCCAATTCTTGTAATAAAT	93.3%	$(ATAG)_5$	4	113-133	100%
STR14	F: CTCTGTGGATAAACAATGAACTGTG<br>R: ACCCTGAGAAGAATTACCATTCTG	100%	$(GAAG)_{11}$	3	291-303	100%
STR15	F:TTCCTACATATTATGCACTACTGGG<br>R:CTTAATGGAAAAGATTTCAGTTCCT	100%	$(ATCT)_{11}$	3	316-332	100%
STR16	F:TTAGGCACTGTTTTCAGTAGCATT<br>R:GAGAAGTGAGTCAGGAGCCTTCTA	100%	$(TCTT)_{13}$	3	216-232	100%
STR17	F:CTGTGAAGGTACAGCATAAATGTGT<br>R:CAGACTACTCCCTAAATGTAAAGCC	93.3%	$(TTTC)_{10}$	4	250-262	100%
STR18	F:GTAGAATCACAGACACTCAGAGTGG<br>R:AATAATACTCCCAGATTTCTCCTCA	100%	$(AGGT)_{10}$	3	179-195	100%
STR19	F: GAACGCTCATTACCTAGGAACTTTA<br>R: CATTTTCTGTCTGTAGGACTGAACA	100%	$(AAAC)_6$	3	308-320	100%
STR20	F: ATCATTAGTCTTTGTAGTACCAGGG<br>R: GTATCATATCCTCCTTATCCGTCT	46.7%	$(AAGG)_7$	3	193-213	66.7%
STR21	F: TGGAAAGTATTTGGACAATAGGTAA	46.7%	$(TTTG)_{12}$	3	247-267	33.3%

	R: GTTTCTCTTCGTCTCTCCTTCAG					
STR22	F: CAGAAGTGCATGAGAGACAGAATAG<br>R: AAATAAGTTTACAGAAGGAGCATCG	40%	(CCCA)$_{22}$	3	108-128	33.3%
STR23	F:AGAACTATAGGTTCTTTGCCTCTTG<br>R:CCTTTGGTATTTATCTGGAGAGTGA	40%	(AAAC)$_{11}$	2	131-139	33.3%
STR24	F:ATCCTGCTAACATCTAATATTCCAA<br>R:TTGCTTCTTTTAAATAGCAAGTGTT	33.3%	(ATATA)$_{6}$	3	211-226	66.7%
STR25	F:ATAATCTCTCTCCATAAACACATGC<br>R:ACAGGAATCTTAACCATGTAATGTG	33.3%	(TCTA)$_{11}$	4	203-243	33.3%
STR26	F:GAAGAAACCAAGTCCAAAACTAAAG<br>R: ACAGTATTGGACTGTGAACAAGTGA	46.7%	(TCTA)$_{6}$	3	266-278	33.3%
STR27	F:CACATTTTTATCCGTTTTATTCACT<br>R:TAATTATGATACAAAGGGAATTTGG	40%	(TCTA)$_{6}$	2	213-225	66.7%
STR28	F:AACTACTGAGCCCATGTGCCTA<br>R:GCCTCTTCCATTTTCTGTGACTC	46.7%	(AATT)7	3	246-286	66.7%
STR29	F:AGGATCTGAAAGCAGCTCATTAT<br>R:GCATTAGCAGGCAGATTGTCA	20%	(AAAG)$_{6}$	2	231-239	66.7%
STR30	F:CAATGCAGGGGATGCAGGTT<br>R:TTTTCTCAGGGTTTACGCCCA	86.7%	(TCTA)$_{10}$	2	254-258	66.7%
STR31	F:TCAATTATCCTTATCCCCACTTG<br>R:CTGTACATTTAGACCCAGCCCT	40%	(TATG)$_{11}$	3	250-270	66.7%
STR32	F:TTTTAAATCACCAAAGATTATTTGG<br>R:ATTTCCAAACAGACAACTGAGGT	20%	(ATAG)$_{5}$	2	281-285	66.7%
STR33	F:TCCTCTTATTTTTCTATTGTCCCTT<br>R:TTTGATGATGATTACCTTCCTTTTA	40%	(GAAG)$_{11}$	2	176-180	66.7%
STR34	F:CACACTCACCTATCCATCAACCA<br>R:CTGATCATTTTCTAGGTCCCTGG	46.7%	(ATCT)$_{11}$	3	469-489	66.7%
STR35	F:TCAAGAAGAGTGCCAAAACATAAG<br>R;CAATGAAAAGCTTTGTCTTCTCTAG	66.6%	(TCTT)$_{13}$	3	204-224	66.7%

STR36	F:GCAATGAGACACAAGTAAGAACAAA<br>R:TGGAACTCTATTGATCACTGAAATC	37.5%	$(TTTC)_{10}$	2	221-225	
STR37	F:TGATTCACTTTGTTATACAGCAGAA<br>R:CCTCTCCAGCATTTATTATTCATAG	46.7%	$(AGGT)_{10}$	4	174-198	
STR38	F:CTTGTTTACAATGGACCGTGC<br>R:CAGACCTAAGGGAGAGCTCCA	46.7%	$(TATT)_{8}$	3	151-171	
STR39	F:CTGGGGTATATGTATTTGCAAGAG<br>R:TGAAATGAATGAATCCTAATCTCAA	66.6%	$(TATC)_{12}$	2	168-172	66.7%
STR40	F:TAATCATGTTAACCCTATCTTCTGG<br>R:GAACTAAGATCCTACAAGCCACAT	40%	$(TATT)_{7}$	3	102-114	
STR41	F:ATGATATTTAAGAGATGCTATGGGA<br>R:GGTTAATCTCCTACCTACTGCAGTT	66.6%	$(TATC)_{12}$	3	195-207	33.3%

Nucleotide Sequences of 19 Tetranucleotide Microsatellite Molecular Markers for the Yangtze Finless Porpoise

STR01：

AAATATGCCTGTAGATGGATGGTTCGATGGTTGGATGTTTGGATACATGGAAGG
ATGGATGGGAGGGAGGAAAGGATGGAGAGAGAGAATAGAT**GATAGATAGATAGAT**
AGATAGATAGATAGATAGATAGATAGATAGATAGATAGATGAGTGAATCAATCAATTAA
ATAGATAAATGCCATACCTTACTTGCATAAAGGAGTTCAACATCTT；

STR02：

AACTAGAAATACACACTGACACCAATCTAGAACTGA**AAAGAAAGAAAGAAAGA**
AAGAAAGAAAGAAAGAAAGAAAGAAAATTGCCCATCTTGAAACATGAACATTGAAT
CTTGATCCTGATTGGAGGTAGAAAAGGATTTCTCCTGAAAA；

STR03：

GCTGTCTCCTACCTTTTAATTATGATTATATAAATATAGATATAGAC**ATCTATCT**
ATCTATCTATCTATCTATCTATCTATCTAATCTCCTCTAAGGTACCTGTAAGGTGCA
TGAGCCCTGAGCCAACACTCCTTTTGGGGATAATGCATGTTTCTGTGTTTCTTT；

STR04：

GCAGAGAAACAAAACCAAAAGGATGTGT**ATAGATAGATAGATAGATAGATAG**
ATAGATAGATAGATAGATAGATAAAGAGAGTCAGTGAAAGTAAGAGGGGGCGGTA
GGGAGAGGGAGAGATGGATTTTAAGGA；

STR05：

AGAAAAACAGAACCAACAGGAGATAGGTGATAGATAAATAGATAGATAGGTAA
GTAGATAGATGGATGGATG**GATAGATAGATAGATAGATAGATAGATAGATAGATA**
GATGGATAATGAAACCTTTGAGCCAACCTGGGCTCTTTGGCTCTGTTGCTCTGGGCAA
CTCTGTGTATGTTGGGAATATTAACATCCACCAACTAT；

STR06：

TAAAAGAGACCAGACCATCTAGTCCAGAGGCTTAAAT**AAGAAAGAAAGAAAGA**
AAGAAAGAAAGAAAGAAAGAAAGAAAGAAAAAAAAAGTTCTTTTCCACCTCTGAA
CTTCTTTTTGATGGAAAGCTGATCTGTTAGGTCCAATATGTA；

STR07：

CTTGACAGATTGGAAATTAAAAACAAAATCAACTAGGCCTTCCCTACAGATCATT

TCAGAATTATTACC**CTATCTATCTATCTATCTAT**CTCTATCTGTTCTGATTTTCTCTA GATCTTTTTCCTACGTGAGAAACGACTTCAGCTAGCATTAAA;

STR08:

ATCAATCAGTCAGCTACCTATCCACTCATCCATCTATTTATCATATTTATCATCTG TCATCTATCTATTTTATCTCCCTACCTTCTATATCTATCTCTCTCTTCTATCTCTCTCCCT TTATCTATCCATCATCTATCATCTGTCTGTCTATATCATCTATCATCTATTATCTATCTA TCTATCATCTATCTATCTTCTATCTATCTATCTTCTATCTATCTATCATCTATCT**TCTAT CTATCTATCTATCTATCTA**TCATCTATCATCTCATCTATCTCCCCCACTAGAATACAA GCTCTATAAGTTCATGACCCT;

STR09:

GTTGAATGTTGGCCTAAGTAATGACCAGAAATCTCACT**GGCAGGCAGGCAGGC AGGCAGGCAGGCAGGCAGGCA**GGCTGGTAGGCAGGCTTCCAAGTCTCATGTGCCTC TGATTT;

STR10:

CTACTGAGCCTGTTCTCTAGAGCCCGTGAGCCATAACTACCGAAGCTTATGCGCC TAGAGCCCATGCTCCGCAACAAGCCACCACAATAAGTAGCCCACGCACTGCAGTGAA GAGTAGCCCCTGTTTGCCGCAACTAGAGAAAGCCCGCATGCAGCAACGAAGACCCAA CACAGCCAAAAAT**AAAGAAAGAAAGAAAGAAAGAAAGAAAAG**AAAACAAAAAGG GTCAACTGTACTTTTACAGAAGAATAAATTATCATTTATATTCAGAACATGTATGTAA CTGATATATCTGCCTGGTTATATAGAAAGGTAAGATTTCTTTCTGCC;

STR11:

GCTTTCTTGGTTTTACAGATTACAGATTGCAAATTGTGATACTTCTCAACCTCATA ATTGTGTGAGTCAATTCCTATAATAAATC**TCTATCTATCTATCTATCTATCTATCTA TCTATCTATCTA**TCATCTCTCCTTATATATTTCCTAATGGTTCTGTTTCTCTG;

STR12:

TCATTAACCTGCATAAGGGTCCTGTGATGTAGGGATTAACCTCCCAATTT**TATGT ATGTATGTATGTATGTATGTATGTATGTATGTATGTATG**GCTGCACTGGGTCCTCG TTGCTGTGCACGAGCTTTCTCTAGTTGCGGCGAGTGGGGGCTACTCTTCGTTGTGGTG CATG;

STR13:

TGTGTGTATTAGTCAGTGTTCTCCAGAGAAACAGAACCAATAGGAAATTAGATA**ATAGATAGATAGATAGATAG**GTAGATAGATAGATTAGATTTATTACAAGAATTGGCTCACAT;

STR14:

CTCTGTGGATAAACAATGAACTGTGAAAAAAAACAATTAACTGTACTCTAAAATAGGTGATTTATATATGTGAAATATATCCCAGTAAAGCTGAGGAAAGAAGGAGGGAGGAAGGGAAGGAGGGAAGGAAGGAAAGAAGGAAGGAAGGAAGGACA**GAAGGAAGGAAGGAAGGAAGGAAGGAAGGAAGGAAGGAAG**GAGGCAGGGAGGAAGGGAGGGAAGAAGGAAGGAATGAAAGATACAGATCTGAAGTGAAGTGTCTCACCCAGAATGGTAATTCTTCTCAGGGT;

STR15:

TTCCTACATATTATGCACTACTGGGACAGTTCCTTCCTTCCTTCCATCTATC**ATCTATCTATCTATCTATCTATCTATCTATCTATCTATCTATCT**ATCAACTGGTTGGCCAAAAGTGCCTTCACTTTTTAAGTAAAAATAAAAGACACATTTTTCATTTTCACCAAGAACTTTATTGGACAACGTATTCACCCTTTTGTCCCACTACCATCTGCCGTTTTTCAGGCAACTTCATGATTCCATCTTCCCAAAACTTTTTATCTTTTTGAGCAAAGAACTGTTCCGGGTGCCTTTTACAGTTTTCCAAGGAACTGAAATCTTTTCCATTAAG;

STR16:

TTAGGCACTGTTTTCAGTAGCATTGCTATGGATAGTAATGTTTTCTTTCTTTCTCTCTCTCTTTCTC**TCTTTCTTTCTTTCTTTCTTTCTTTCTTTCTTTCTTTCTTTCTTTCTTTCTT**ATCTTCATCTTCCAGTTTTTCTAATTATTTCATATGAAATGAATAATCCCATGGTCCAATCCCCAGTCATCCATAGAAGGCTCCTGACTCACTTCTC;

STR17:

CTGTGAAGGTACAGCATAAATGTGTGAAAATAAAATATCTCCTGACCTCGCCATCAGAGACATAGATTTTATTTTTTTCTTAGGAGTGTCTGGTGAGTTTTCTTTCTTTCTTTCCTTTCTTTCTCTTAT**TTTCTTTCTTTCTTTCTTTCTTTCTTTCTTTCTTTCTTTC**CTCTCTCTCTCTCTCTTTCTTTCTTCTTCTCCCTTTTTTTTGGTGGTGGTTGTCGCAGCTCAACGGGGCTTTACATTTAGGGAGTAGTCTG;

STR18:

GTAGAATCACAGACACTCAGAGTGGTGAGGGCAGGAACTCTGCCAGGGAGCCCT

GGGCCGGGGCAAGGATGTCCTTCCTGCTACCACCACAGGGAGGG**AGGTAGGTAGGT AGGTAGGTAGGTAGGTAGGTAGGTAGGT**AGCTGGCTGTCTCCTCTCATATCCTTGG CACATGAGGAGAAATCTGGGAGTATTATT;

STR19:

GAACGCTCATTACCTAGGAACTTTAATTAATCTAGTTGAACTTAAGCTTAAAGAA ATAATTGTTGGGGCTTCCCTGGTGGCGCAGTGGTTGGGAGTCCGCCTGCCGATGCAGG GGACACGGGTTCGTGCCCCGGTCTGGGAGGATCCCACATGCCGCGGAGCGGCTGGGC TCGTGAGCCATGGCCGCTGAGCCTGCGCGTCCGGAGCCTGTGCTCCACAACGGGAGA GGTCACAACAGTGAGAGGCCCGCGTACCGCAAA**AAACAAACAAACAAACAAACAA AC**AAAAAAAAAAAGAAATAATTGTTCAGTCCTACAGACAGAAAATG
